# Comparison of Cardiovascular Risk Factors and Outcomes Among Practicing Physicians vs the General Population in Ontario, Canada

**DOI:** 10.1001/jamanetworkopen.2019.15983

**Published:** 2019-11-22

**Authors:** Dennis T. Ko, Anna Chu, Peter C. Austin, Sharon Johnston, Brahmajee K. Nallamothu, Idan Roifman, Natasa Tusevljak, Jacob A. Udell, Erica Frank

**Affiliations:** 1 ICES, Toronto, Ontario, Canada; 2 Schulich Heart Centre, Sunnybrook Health Sciences Centre, Toronto, Ontario, Canada; 3 Department of Medicine, University of Toronto, Toronto, Ontario, Canada; 4 Bruyère Research Institute, Ottawa, Ontario, Canada; 5 Departmentof Family Medicine, University of Ottawa, Ottawa, Ontario, Canada; 6 Division of Cardiovascular Diseases, Department of Internal Medicine, University of Michigan, Ann Arbor; 7 Women’s College Hospital, Toronto, Ontario, Canada; 8 School of Population and Public Health, University of British Columbia, Vancouver, British Columbia, Canada

## Abstract

**Importance:**

Although cardiovascular disease is the leading cause of death in most developed countries, little is known about current physicians’ cardiovascular health and outcomes.

**Objective:**

To compare cardiac risk factor burden, health services use, and major cardiovascular event incidence between physicians and the general population.

**Design, Setting, and Participants:**

This cohort study used data from practicing physicians and nonphysicians without cardiovascular disease aged 40 to 75 years in Ontario, Canada. Cohorts were assembled beginning January 1, 2008, and were followed up to December 31, 2015. Data analysis was performed between November 2017 and September 2019.

**Exposure:**

Being a practicing physician.

**Main Outcomes and Measures:**

The primary outcome was 8-year incidence of a major cardiovascular event (ie, cardiovascular death or hospitalization for myocardial infarction, stroke, heart failure, or coronary revascularization). Secondary outcomes included health services used, such as physician assessments and guideline-recommended tests.

**Results:**

The cohort comprised 17 071 physicians (mean [SD] age, 53.3 [8.8] years; 11 963 [70.1%] men) and 5 306 038 nonphysicians (mean [SD] age, 53.7 [9.5] years; 2 556 044 [48.2%] men). Physicians had significantly lower baseline rates of hypertension (16.9% vs 29.6%), diabetes (5.0% vs 11.3%), and smoking (13.1% vs 21.6%), while having better cholesterol profiles (total cholesterol levels >240 mg/dL, 13.3% vs 16.5%; low-density lipoprotein cholesterol >130 mg/dL, 33.2% vs 36.8%); age- and sex-adjusted differences were even larger. Physicians also had lower rates of periodic health examinations (58.9% [95% CI, 57.5%-60.4%] vs 67.9% [95% CI, 67.8%-67.9%]), hyperlipidemia screening (76.3% [95% CI, 74.7%-78.0%] vs 83.8% [95% CI, 83.7%-83.9%]), and diabetes screening (79.0% [95% CI, 77.3%-80.8%] vs 85.3% [95% CI, 85.2%-85.4%]), but higher rates of cardiologist consultations (25.2% [95% CI, 24.2%-26.3%] vs 19.5% [95% CI, 19.4%-19.5%]). The 8-year age- and sex-standardized primary outcome incidence was 4.4 major cardiovascular events per 1000 person-years for physicians and 7.1 major cardiovascular events per 1000 person-years for the general population. After adjusting for age, sex, socioeconomic status, and cardiac risks and comorbidities, physicians had a 22% lower hazard (hazard ratio, 0.78; 95% CI, 0.72-0.85) of experiencing the primary outcome compared with the general population.

**Conclusions and Relevance:**

Practicing physicians in Ontario had fewer cardiovascular risk factors, underwent less preventive testing, and were less likely to experience major adverse cardiovascular outcomes than the general population.

## Key Points

**Question:**

How do cardiovascular health outcomes of physicians compare with those of the general population?

**Findings:**

This cohort study of 17 071 practicing physicians and 5 306 038 members of the general population in Ontario found that physicians used fewer guideline-recommended preventive services and had lower rates of cardiac risk factors. At 8 years’ follow-up, physicians had a substantially lower risk of adverse outcomes than the general population, even after adjusting for differences in risk factors and health services.

**Meaning:**

Ontario physicians have better cardiovascular outcomes than the general population, and the difference in outcomes between physicians and nonphysicians was not fully explained by traditional cardiac risk factors.

## Teaser

This cohort study compares cardiac risk factor burden, health services use, and major cardiovascular event incidence between physicians and the general population in Ontario, Canada.

## Introduction

Cardiovascular disease is the leading cause of death in most developed countries. Moreover, cardiovascular death has been shown to be the leading cause of death among physicians in the United Kingdom and United States.^1,2^ Although physicians’ personal health practices are generally good,^3,4^ they often favor their professional obligations over their own health.^5,6^ As more demands are placed on physicians’ time in current medical practice, there is an increasing recognition that physicians are likely to be fatigued, stressed, or burned out.^6,7,8,9^ Unwell physicians can also potentially adversely affect the health care system by reducing quality of care and jeopardizing patient safety.^7^ Older studies have suggested that physicians have longer life expectancies and lower cardiovascular mortality rates compared with the general population, but the lack of in-depth knowledge about physicians’ risk factors and comorbidities limits the understanding of overall physician cardiovascular health.^1,2,10^ Furthermore, those studies^1,2,10^ enrolled cohorts from more than 2 decades ago, and little is known about physicians’ overall cardiovascular health and outcomes currently. Accordingly, the main objective of this study was to compare cardiac risk factors, health services use, and cardiovascular outcomes among practicing physicians with those of the general population in Ontario, Canada. We hypothesized that physicians would have lower rates of cardiovascular outcomes compared with the general population.

## Methods

### Data Sources

The Cardiovascular Health in Ambulatory Care Health Research Team (CANHEART) cohort was created from 9 798 473 million adults living in Ontario.^11,12,13^ Nineteen population-based health databases of Ontarians, including hospitalization records, physician claims, laboratory tests, surveys, and vital statistics, were linked with unique encoded personal identifiers and analyzed at ICES (formerly known as the Institute for Clinical Evaluative Sciences).^11,12,13^ Data sources included the Ontario Registered Persons Database, a registry of Ontario residents with Ontario’s universal health insurance coverage; Canadian Institute for Health Information Discharge Abstract Database, Ontario Diabetes Database,^14^ Ontario Hypertension Database,^15^ and Ontario Cancer Registry, to identify hospitalizations, baseline cardiac risk factors, and comorbidities; Registrar General of Ontario Database, to determine cause of death; Canadian Community Health Surveys from 2001 to 2012, to assess lifestyle factors; Ontario Health Insurance Plan Physician Claims database, to capture clinical assessments and cardiac testing delivered; Dynacare Medical Laboratories database, to determine cholesterol levels; and the College of Physicians and Surgeons of Ontario registry, for demographic characteristics, specialty, medical school locations, and year of graduation of all practicing physicians in Ontario.

This cohort study has been approved by the research ethics board at Sunnybrook Health Sciences Centre, Toronto, Canada. The need for informed consent is not required for this study under Ontario’s health information privacy law. This study follows the Strengthening the Reporting of Observational Studies in Epidemiology (STROBE) reporting guideline.

### Study Sample

To be consistent with prior studies of primary prevention and cardiovascular events,^16^ we restricted the study sample to individuals aged 40 to 75 years without a history of hospitalization for acute myocardial infarction (MI), stroke, heart failure, or coronary revascularization (percutaneous coronary intervention or coronary artery bypass grafting). We applied the inclusion criteria to individuals who were alive on January 1, 2008, which served as the index date of outcomes. Follow-up for outcomes continued until the study end date of December 31, 2015, time of death, or loss of Ontario health insurance coverage. All individuals were Ontario residents, had a valid Ontario Health Card number, and did not reside in a long-term care facility or nursing home. Individuals registered with the College of Physicians and Surgeons of Ontario at the time of cohort inception were considered to be in the physician group.

### Health Services Use

Health services use pertaining to cardiovascular health during the 8 years after cohort inception was evaluated. Assessments by primary care physicians and specialists, periodic health examinations, and screening for cholesterol and diabetes were evaluated using Ontario Health Insurance Plan fee codes. Canadian practice guidelines currently recommend cholesterol testing every 5 years and diabetes screening every 3 years for all individuals aged 40 years or older.^17,18^ The use of cardiac diagnostic testing, including electrocardiograms, echocardiography, stress testing, and cardiac catheterization, was also determined. Any assessment during the study entire study period was considered as being performed.

### Cardiovascular Outcomes

Our primary clinical outcome was a composite of cardiovascular death and hospitalization for acute MI, stroke, heart failure, or coronary revascularization (percutaneous coronary intervention or coronary artery bypass grafting) at 8 years (eTable 1 in the Supplement). Cardiovascular death was assessed using the vital statistics database, whereas hospitalization and revascularizations were determined using Canadian Institute for Health Information databases. Complete follow-up of all end points was available up to December 31, 2015.

### Statistical Analysis

Demographic and clinical characteristics of the physician group were compared with the general population using χ^2^ tests for categorical variables and 1-way analysis of variance for continuous variables. Health services utilization and outcome rates were standardized by age and sex using the 2006 Ontario Census population as the reference population, because this was the census year closest to our cohort inception date. Incidence of the primary outcome was calculated in the overall cohort and prespecified subgroups of physicians on the basis of age, sex, neighborhood income quintile, and specialty. All clinical specialties were grouped into 10 main types (anesthesiology, cardiology, family medicine, internal medicine, laboratory, obstetrics and gynecology, pediatrics, psychiatry, radiology, and surgery), as shown in eTable 2 in the Supplement.

Clinical outcomes among physicians vs the general population were compared using cause-specific hazards models that accounted for the competing risk of noncardiovascular death. Factors included in the models were selected using clinical knowledge and included demographic characteristics, socioeconomic status, cardiac risk factors (smoking, hypertension, diabetes, and cholesterol levels), comorbidity score using the John Hopkins Adjusted Clinical Groups, and use of health services (physician assessments).^19,20^ We used income quintile as a surrogate of socioeconomic status, estimated by calculating the mean neighborhood household income using postal codes of the individuals, adjusted for household size and housing costs. Multiple imputation methods were used to impute missing data, such as smoking status and cholesterol levels, as described elsewhere.^14,15^

Several additional analyses were performed to ensure that our results were robust. First, we adjusted for ethnicity of the individuals using a previously validated surname algorithm. The surname algorithm has been compared with self-identified ethnicity with positive predictive values of 89.3% for South Asian names and 91.9% for Chinese names.^21^ Second, we also accounted for the rural residency of individuals because it could be associated with access to care and outcomes. Third, among individuals who were older than 65 years, we also accounted for the use of statin medications and antihypertensive medications at baseline.

To explore whether factors such as demographic and cardiac risk factors had similar associations with adverse cardiovascular outcomes among physicians compared with the general population, we performed a stratified analysis by generating cause-specific hazard models separately among physicians and the general population. We also conducted a series of cause-specific hazard models by entering each group of factors (demographic characteristics, socioeconomic status, cardiac risk factors, comorbidity, and health services) in a sequential manner to explore reasons for the difference in outcomes between groups.

All analyses were conducted at ICES using SAS statistical software version 9.4 (SAS Institute) between November 2017 and September 2019. All *P* values were from 2-sided tests, with *P* < .05 considered statistically significant.

## Results

### Study Cohort

The Figure details the creation of the study cohort. A total of 9 798 473 community-dwelling adults had a valid health card in Ontario on January 1, 2008. We excluded 4 222 268 individuals aged younger than 40 years or older than 75 years, and 253 096 patients with histories of MI, stroke, heart failure, or coronary revascularization. This resulted in 17 071 physicians and 5 306 038 nonphysicians in the study population. The total follow-up time for physicians and the general population was 134 610 and 41 008 319 person-years, respectively. The mean (SD) follow-up time was 7.9 (0.7) years for physicians and 7.7 (1.1) years for the general population. There were 1.3% of physicians and 2.7% of the general population who were lost to follow-up because of the loss of Ontario health insurance eligibility during the study period.

**Figure.**
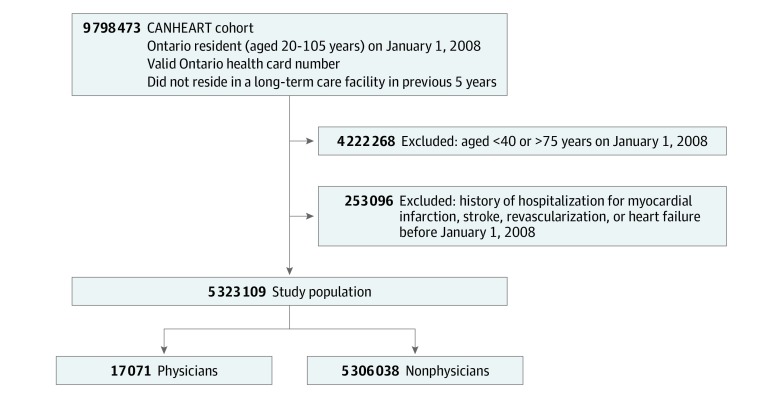
Cohort Creation Flowchart shows exclusion criteria for the Cardiovascular Health in Ambulatory Care Health Research Team (CANHEART) cohort.

### Baseline Characteristics, Cardiac Risk Factors, and Comorbidities

Physicians in Ontario had a similar mean (SD) age as the general population (53.3 [8.8] years vs 53.7 [9.5] years), but were more likely to be men (70.1% [11 963] vs 48.2% [2 556 044]), reside in high-income neighborhoods (61.0% [10 380] vs 21.5% [1 137 565]), and be long-term residents of Canada (93.1% [15 893] vs 85.6% [4 542 423]) (all *P* < .001) (Table 1). Most physicians practicing in Ontario (72.1%) were Canadian medical graduates. The most common physician specialty was family medicine (47.0%), followed by noncardiology internal medicine specialties (13.1%), and surgical specialties (10.0%). Cardiologists accounted for 2.3% of the physician cohort.

**Table 1.** Demographic and Baseline Characteristics of Physicians and the General Population

Variable	No. (%)		*P* Value
	Physicians (n = 17 071)	General Population (n = 5 306 038)	
Demographic characteristics			
Age on January 1, 2008, mean (SD), y	53.3 (8.8)	53.7 (9.5)	<.001
Male	11 963 (70.1)	2 556 044 (48.2)	<.001
Highest neighborhood income quintile	10 380 (61.0)	1 137 565 (21.5)	<.001
Rural residence	1132 (6.6)	673 276 (12.7)	<.001
Immigrant status			
Long-term resident	15 893 (93.1)	4 542 423 (85.6)	<.001
Immigrated to Canada, y			
10-20	674 (3.9)	435 168 (8.2)	
<10	504 (3.0)	328 447 (6.2)	
Ethnicity			
Non-Chinese and non–South Asian	15 306 (89.7)	4 898 898 (92.3)	<.001
Chinese	1078 (6.3)	263 540 (5.0)	
South Asian	687 (4.0)	143 600 (2.7)	
Cardiac risk factors			
Hypertension	2887 (16.9)	1 568 382 (29.6)	<.001
Diabetes	855 (5.0)	599 548 (11.3)	<.001
Smoker^a^	1708 (13.1)	1 075 275 (21.6)	.03
Cholesterol levels, mg/dL^b^			
Total cholesterol			
Mean (SD)	197.3 (38.7)	202.6 (40.2)	<.001
>240 mg/dL	451 (13.3)	219 004 (16.5)	<.001
Low-density lipoprotein cholesterol			
Mean (SD)	115.9 (34.0)	119.5 (34.8)	<.001
>130 mg/dL	1082 (33.2)	465 594 (36.8)	<.001
High-density lipoprotein cholesterol, mg/dL, mean (SD)	58.7 (16.6)	56.7 (16.2)	<.001
Comorbidities			
Atrial fibrillation	304 (1.8)	59 193 (1.1)	<.001
Cancer	824 (4.8)	249 699 (4.7)	.46
Chronic obstructive pulmonary disease	293 (1.7)	417 470 (7.9)	<.001
Asthma	797 (4.7)	539 040 (10.2)	<.001
Johns Hopkins ACG score, mean (SD)^c^	6.0 (4.0)	8.0 (4.4)	<.001

Abbreviation: ACG, Adjusted Clinical Groups.

SI conversion factors: to convert total cholesterol, high-density lipoprotein cholesterol, and low-density lipoprotein cholesterol to mmol/L, multiply by 0.0259.

^a^ Data are from a subpopulation of respondents to the 2001 to 2012 Canadian Community Health Surveys (185 physicians and 94 802 members of the general population). Information on smoking was available for 1.8% of individuals who completed the Canadian Community Health Survey.

^b^ Data are from a subpopulation of individuals with test results from Dynacare Medical Laboratories between 2002 and 2007 (3395 physicians and 1 326 939 members of the general population). Where multiple cholesterol values were available, we used the result closest to January 1, 2008. Fasting cholesterol values were recommended at the time of the study. Information on lipid results was available for 25.0% of the study population who completed cholesterol testing.

^c^ The Johns Hopkins ACG score is a measure of comorbidity based on a patient’s age, sex, and diagnosis codes from ambulatory and in-patient care settings in the previous 5 years, and was determined using The Johns Hopkins ACG System Version 7. The range of possible scores is 0 to 32, with lower scores indicating fewer comorbid conditions.

Physicians had substantially lower baseline rates of cardiac risk factors compared with the general population, including hypertension (16.9% [2887] vs 29.6% [1 568 382]), diabetes (5.0% [855] vs 11.3% [599 548]), and smoking (13.1% [1708] vs 21.6% [1 075 275]) (Table 1). Fewer physicians had total cholesterol levels greater than 240 mg/dL (13.3% [451] vs 16.5% [219 004]) and low-density lipoprotein cholesterol greater than 130 mg/dL (33.2% [1082] vs 36.8% [465 594]) (to convert total cholesterol and low-density lipoprotein cholesterol to millimoles per liter, multiply by 0.0259). Physicians also had slightly higher rates of atrial fibrillation (1.8% [304] vs 1.1% [59 193]), similar rates of cancer (4.8% [824] vs 4.7% [249 699]), and lower rates of chronic obstructive pulmonary disease (1.7% [293] vs 7.9% [417 470]) and asthma (4.7% [797] vs 10.2% [539 040]) compared with the general population.

### Use of Health Care Services

Age- and sex-standardized rates of ambulatory health care services are shown in Table 2. Over the course of 8 years, physicians had lower rates of being assessed by a primary care physician (88.1% [95% CI, 86.4%-89.9%] vs 93.6% [95% CI, 93.5%-93.7%]) and significantly fewer visits per year (mean, 1.7 [95% CI, 1.7-1.8] vs 4.0 [95% CI, 4.0-4.0]) compared with the general population. Physicians also had fewer periodic health examinations (58.9% [95% CI, 57.5%-60.4%] vs 67.9% [95% CI, 67.8%-67.9%]). In contrast, they were more likely to be seen by a specialist (93.6% [95% CI, 91.8%-95.4%] vs 85.4% [95% CI, 85.3%-85.5%]) and by a cardiologist (25.2% [95% CI, 24.2%-26.3%] vs 19.5% [95% CI, 19.4%-19.5%]) in this time frame (all *P* < .001). Unadjusted rates of health services utilization are shown in eTable 3 in the Supplement.

**Table 2.** Use of Health Services Among Physicians and the General Population

Variable	Use Rate, % (95% CI)^a^		*P* Value
	Physicians (n = 17 071)	General Population (n = 5 306 038)	
Physician visits			
Primary care physician	88.1 (86.4-89.9)	93.6 (93.5-93.7)	<.001
Annual visits, mean, No. (95% CI)	1.7 (1.7-1.8)	4.0 (4.0-4.0)	<.001
Any specialist	93.6 (91.8-95.4)	85.4 (85.3-85.5)	<.001
Annual visits, mean, No. (95% CI)	3.0 (2.9-3.1)	2.2 (2.2-2.2)	<.001
Cardiologist	25.2 (24.2-26.3)	19.5 (19.4-19.5)	<.001
Periodic health examination	58.9 (57.5-60.4)	67.9 (67.8-67.9)	<.001
Cardiac risk factor assessment^b^			
Screening			
Cholesterol	76.3 (74.7-78.0)	83.8 (83.7-83.9)	<.001
Diabetes	79.0 (77.3-80.8)	85.3 (85.2-85.4)	<.001
Cardiac testing			
Electrocardiography	64.4 (62.9-65.9)	72.4 (72.3-72.5)	<.001
Echocardiography	34.0 (32.8-35.2)	32.3 (32.2-32.3)	.004
Noninvasive stress test	29.1 (28.0-30.1)	29.7 (29.6-29.7)	.26

^a^ Rates were age- and sex-standardized to the 2006 Ontario Census population.

^b^ Cholesterol screening was calculated among all individuals aged 40 years or older, as recommended by the Canadian Cardiovascular Society. Diabetes screening was calculated among all nondiabetic individuals at baseline and excluded testing during pregnancy.

Physicians underwent less screening for cholesterol (76.3% [95% CI, 74.7%-78.0%] vs 83.8% [95% CI, 83.7%-83.9%]) and diabetes (79.0% [95% CI, 77.3%-80.8%] vs 85.3% [95% CI, 85.2%-85.4%) (*P* < .001 for both) than the general population (Table 2). With regard to cardiac tests, physicians had higher use of echocardiography (34.0% [95% CI, 32.8%-35.2%] vs 32.3% [95% CI, 32.2%-32.3%]; *P* = .004), whereas the general population had higher rates of electrocardiography use (72.4% [95% CI, 72.3%-72.5%] vs 64.4% [95% CI, 62.9%-65.9%]; *P* < .001). The use of noninvasive stress testing was not significantly different between the 2 groups (29.1% [95% CI, 28.0%-30.1%] vs 29.7% [95% CI, 29.6%-29.7%]; *P* = .26).

eTable 4 in the Supplement shows health services use stratified by sex. Differences in health services use between physicians and the general population were larger among men than women. For example, male physicians had an 8.9% lower rate of seeing a primary care physician compared with male nonphysicians, whereas the difference was only 2.2% between female physicians and nonphysicians. Male physicians also had a 15% lower rate of obtaining a periodic health examination than male nonphysicians vs a difference of 3.2% between female physicians and nonphysicians.

### Cardiovascular Events

The 8-year incidence rate of the composite outcome of cardiovascular death or hospitalization for MI, stroke, heart failure, or coronary revascularization was 4.4 per 1000 person-years for physicians and 7.1 per 1000 person-years for the general population (Table 3). After adjustment, physicians still had a 22% lower hazard (hazard ratio [HR], 0.78; 95% CI, 0.72-0.85) of experiencing the primary outcome compared with the general population. Lower hazards of the primary outcome were also seen after adjustment for ethnicity (HR, 0.79; 95% CI, 0.73-0.86), rural residency (HR, 0.80; 95% CI, 0.74-0.87), and use of statins and antihypertensive medication (HR, 0.85; 95% CI, 0.73-0.98). The incidence of cardiovascular death was 52% lower among physicians than the general population (0.72 per 1000 person-years vs 1.5 per 1000 person-years) with an adjusted HR of 0.45 (95% CI, 0.35-0.58; adjusted risk, 55% lower). Physicians also had a significantly lower incidence and risk of MI (adjusted risk, 32% lower) and stroke (adjusted risk, 27% lower).

**Table 3.** Adjusted HRs of Cardiovascular Outcomes Comparing Physicians and the General Population^a^

Outcomes	Incidence per 1000 Person-Years (No. of Events)		HR (95% CI)
	Physicians	General Population	
Cardiovascular death, myocardial infarction, stroke, heart failure, or coronary revascularization	4.4 (602)	7.1 (249 666)	0.78 (0.72-0.85)
Cardiovascular death	0.7 (64)	1.5 (50 704)	0.45 (0.35-0.58)
Myocardial infarction, stroke, or heart failure	2.2 (300)	4.3 (153 900)	0.66 (0.59-0.74)
Myocardial infarction	1.2 (181)	2.2 (83 169)	0.68 (0.58-0.78)
Stroke	0.8 (97)	1.4 (48 245)	0.73 (0.60-0.89)
Coronary revascularization	2.7 (422)	3.6 (130 727)	0.94 (0.86-1.04)

Abbreviation: HR, hazard ratio.

^a^ Incidence rates are age- and sex-standardized to the 2006 Ontario census population. Hazard ratios are adjusted for demographic characteristics (age and sex), socioeconomic status (as measured by neighborhood income quintile), cardiac risk factors (smoking, hypertension, diabetes, and cholesterol levels), comorbidity score, and use of health services (physician assessments). General population was the reference group.

By age, sex, neighborhood income quintile, and specialty, we found significantly lower incidence rates of the primary composite cardiovascular events among physicians compared with nonphysicians (eTable 5 in the Supplement). There was variation in the primary outcome event rate across physician specialties, but when comparing outcomes of specialists with family physicians as a reference, outcome rates were not significantly different (data not shown).

### Factors Associated With Cardiovascular Outcomes Among Physicians and the General Population

We assessed the importance of demographic and cardiac risk factors in the development of cardiovascular outcomes among physicians and the general population. An income gradient showing progressively higher risk with lower income quintile was seen only among the general population (HR, 1.32 [95% CI, 1.30-1.33] for the lowest income quintile vs 1.08 [95% CI, 1.07-1.10] for the fourth income quintile compared with the highest income quintile) (Table 4). For cardiac risk factors, HRs for the primary outcome were lower for physicians compared with the general population for hypertension (HR, 1.28 [95% CI, 1.05-1.55] vs 1.50 [95% CI, 1.48-1.51]) and smoking (HR, 1.15 [95% CI, 0.85-1.55] vs 1.50 [95% CI, 1.48-1.53]). In adjusted models comparing physicians and nonphysicians, significant interactions were observed for hypertension (HR, 1.22 [95% CI, 0.98-1.52] vs 1.50 [95% CI, 1.48-1.52]; *P* = .03) and for smoking (HR, 1.11 [95% CI, 0.81-1.51] vs 1.50 [95% CI, 1.47-.154]; *P* = .049).

**Table 4.** Factors Associated With Cardiovascular Outcomes in Physicians and the General Population

Factor	Physicians		General Population	
	HR (95% CI)	*P* Value	HR (95% CI)	*P* Value
Participants, No.	17 071		5 306 038	
Demographic characteristics				
Age on January 1, 2008, per y	1.07 (1.06-1.08)	<.001	1.06 (1.06-1.07)	<.001
Female	0.35 (0.26-0.47)	<.001	0.46 (0.45-0.46)	<.001
Income quintile^a^				
1	0.94 (0.68-1.28)	.68	1.32 (1.30-1.33)	<.001
2	0.97 (0.79-1.36)	.88	1.19 (1.17-1.20)	<.001
3	1.03 (0.79-1.35)	.81	1.14 (1.12-1.15)	<.001
4	0.95 (0.76-1.20)	.68	1.08 (1.07-1.10)	<.001
Cardiac risk factors				
Hypertension	1.28 (1.05-1.55)	.01	1.50 (1.48-1.51)	<.001
Diabetes	1.56 (1.20-2.03)	<.001	1.67 (1.66-1.69)	<.001
Smoker	1.15 (0.85-1.55)	.38	1.50 (1.48-1.53)	<.001
Cholesterol (per mmol/L)				
Total	1.06 (0.96-1.18)	.27	1.07 (1.06-1.07)	<.001
High-density lipoprotein	0.73 (0.58-0.93)	.01	0.75 (0.74-0.76)	<.001
Johns Hopkins ACG score^b^	1.00 (0.97-1.03)	.96	1.02 (1.01-1.02)	<.001

Abbreviations: ACG, Adjusted Clinical Groups; HR, hazard ratio.

^a^ The fifth quintile is the highest income level and is the reference against which the other levels are compared (HR = 1; not shown).

^b^ The Johns Hopkins ACG score is a measure of comorbidity based on a patient’s age, sex, and diagnosis codes from ambulatory and in-patient care settings in the previous 5 years, and was determined using The Johns Hopkins ACG System Version 7. The range of possible scores is 0 to 32, with lower scores indicating fewer comorbid conditions.

### Sequential Adjustment to Explore Difference in Outcomes Among Physicians and the General Population

eTable 6 in the Supplement shows sequential adjustment for age, sex, neighborhood income quintile, traditional cardiac risk factors, comorbidity score, and health services use to explore the extent to which they could explain the outcome difference between physicians and the general population (Table 4). The age-and sex-adjusted HR increased from 0.62 (95% CI, 0.57-0.67) to 0.68 (95% CI, 0.63-0.74) after additional income adjustment, to 0.78 (95% CI, 0.72-0.85) after cardiac risk factors adjustment, to 0.80 (95% CI, 0.74-0.87) after comorbidity adjustment, and to 0.78 (95% CI, 0.72-0.85) after health services use adjustment.

## Discussion

We found that physicians had lower baseline rates of traditional cardiac risk factors and were less likely to visit a primary care physician or undergo guideline-recommended screening tests, but were more likely to consult with a specialist compared with the general population. Even after accounting for these differences, physicians had considerably lower adjusted risks of experiencing adverse cardiovascular outcomes compared with the general population: 55% lower for cardiovascular death, 32% lower for MI, and 27% lower for stroke. In addition, physicians’ lower cardiovascular risk persisted even after adjustment for traditional factors associated with adverse cardiovascular outcomes, suggesting the need to further evaluate unconventional factors that could be contributing to better outcomes among physicians. Given the potential of residual confounding in observational studies, additional studies are also needed to understand the reasons for these differences.

Few contemporary studies have evaluated in depth the cardiovascular risk factors and outcomes of physicians compared with the general population using routinely collected data. In the 1990s, data from the US Women Physician’s Health Study found that female physicians were less likely to smoke or binge drink and more likely to exercise and eat more fruits and vegetables than were other women.^4^ Likewise, more than 90% of Canadian physicians report being in good to excellent health (vs 70% of Canadians aged 20 to 34 years), only 3% smoked cigarettes (vs 18% of other women and 15% of other men), they ate more fruits and vegetables, exercised more (averaging 4.7 hours per week), and admitted binge-drinking markedly less than other Canadians.^3^ Similar findings of favorable cardiovascular practices have also been shown in electronic medical records of Israeli physicians^22^ and in surveyed US and Colombian medical students.^3,23,24^

We were able to extend these earlier findings by comparing the health of all practicing physicians to that of the entire population in Ontario and found that physicians had about half the rates of hypertension, diabetes, and smoking, while also having a more favorable cholesterol profile with lower low-density lipoprotein and higher high-density lipoprotein cholesterol levels. Given recent concerns of burnout and deterioration of physicians’ health, further examination of the temporal trends of cardiovascular health of physicians would be important.

This study also provides insight into how physicians encounter the health care system. Physicians had fewer visits to family physicians and periodic health examinations, but were more likely than the general population to have consulted with specialists. This discrepancy could be associated with physicians engaging more in routine self-care and therefore not routinely seeing or consulting with a primary care physician as often as the general population.^7,25,26^ In addition, many physicians have encounters with specialists in their work environment that facilitate direct consultations with specialists, enabling bypassing of the traditional primary care gateway.

Although screening for hyperlipidemia and diabetes was recommended for all individuals in our cohort,^17,18^ we found that screening rates were significantly lower among physicians compared with nonphysicians, a finding that is consistent with those of other studies.^27,28^ In addition, we also observed sex-based differences in that male physicians had the lowest rates of primary care physician visits, periodic health examinations, and cholesterol and diabetes screening compared with male nonphysicians and women. If physicians are foregoing preventive screenings, both their and their patients’ health may be harmed, because there is an association between physicians’ and patients’ screening and primary prevention practices.^3,4,23,24,29^

A remarkable finding was that, even though we accounted for the difference in cardiac risk factors and health services practices, physicians had a substantially lower hazard of major cardiovascular events compared with nonphysicians. Although we do not understand this observation fully, several factors could be contributory. First, it is possible that physicians had more awareness of their cardiac symptoms and made earlier diagnoses. For example, we observed that physicians had higher rates of atrial fibrillation, even though their anticipated rate of atrial fibrillation should be much lower because of their lower rates of hypertension. Second, in examining the association between risk factors and outcomes, we found that the HRs for hypertension and smoking were closer to unity for physicians. This observation of a lower association of known cardiac risk factors among physicians than nonphysicians potentially suggests that physicians engage in better management of risk factors (or prevention of their development).

### Limitations

Several limitations of our study merit discussion. First, we compared data for physicians with those for the general population and did not compare them with those for other professional groups of high socioeconomic status because of the lack of data availability. Although we accounted for socioeconomic status using income quintile in our analyses, it is possible that lower event rates seen among physicians may be associated with factors such as education, occupation, or other social factors. Second, although it has been shown that patients hospitalized with MI and heart failure have similar characteristics between Canada and the United States,^30,31^ it is difficult to know whether the findings of our study could be generalized to the United States because of the different ethnic background of our study cohort and differences in access to primary preventive care between the 2 countries. In addition, we lacked detailed information on the lifestyle habits of physicians and nonphysicians and, thus, could not examine the extent to which they might modify the outcome differences.

## Conclusions

Ontario physicians had fewer cardiovascular risk factors, received fewer cardiovascular preventive services, and were significantly less likely to develop major adverse cardiovascular outcomes than the general population. Future studies are needed to understand the factors that explain these differences.

## Notes

Supplement. **eTable 1.** Exposure and Cardiovascular Outcome Coding Descriptions **eTable 2.** Classification of Physicians Into Specialty Groups **eTable 3.** Crude Rates of Use of Ambulatory Health Services Among Physicians and the General Population **eTable 4.** Use of Ambulatory Health Services Among Physicians and the General Population, Stratified by Sex, 2008-2015 **eTable 5.** Rates of Major Adverse Cardiovascular Events Among Physician Subgroups vs the General Population **eTable 6.** Sequential Adjustment to Explore Factors Associated With Risk of a Major Adverse Cardiovascular Event
