# Supplementary material for: Comparison of Cardiovascular Risk Factors and Outcomes Among Practicing Physicians vs the General Population in Ontario, Canada
Source: JAMA Netw Open. 2019 Nov 22;2(11):e1915983. doi: 10.1001/jamanetworkopen.2019.15983 (PMC6902820; doi:10.1001/jamanetworkopen.2019.15983)
Supplement: Supplement. — eTable 1. Exposure and Cardiovascular Outcome Coding Descriptions eTable 2. Classification of Physicians Into Specialty Groups eTable 3. Crude Rates of Use of Ambulatory Health Services Among Physicians and the General Population eTable 4. Use of Ambulatory Health Services Among Physicians and the General Population, Stratified by Sex, 2008-2015 eTable 5. Rates of Major Adverse Cardiovascular Events Among Physician Subgroups vs the General Population eTable 6. Sequential Adjustment to Explore Factors Associated With Risk of a Major Adverse Cardiovascular Event [file jamanetwopen-2-e1915983-s001.pdf]

Supplementary Online Content

Ko DT, Chu A, Austin PC, et al. Comparison of cardiovascular risk factors and outcomes among practicing physicians vs the general population in Ontario, Canada. *JAMA Netw Open*. 2019;2(11):e1915983. doi:10.1001/jamanetworkopen.2019.15983

eTable 1. Exposure and Cardiovascular Outcome Coding Descriptions

eTable 2. Classification of Physicians Into Specialty Groups

eTable 3. Crude Rates of Use of Ambulatory Health Services Among Physicians and the General Population

eTable 4. Use of Ambulatory Health Services Among Physicians and the General Population, Stratified by Sex, 2008-2015

eTable 5. Rates of Major Adverse Cardiovascular Events Among Physician Subgroups vs the General Population

eTable 6. Sequential Adjustment to Explore Factors Associated With Risk of a Major Adverse Cardiovascular Event

This supplementary material has been provided by the authors to give readers additional information about their work.

© 2019 Ko DT et al. *JAMA Network Open.*

eTable 1. Exposure and Cardiovascular Outcome Coding Descriptions

	Data source	Codes
**Exposure**		
Physician	College of Physicians and Surgeons of Ontario registry	Classified as physician if in registry as of January 1, 2008
**Cardiovascular outcomes**		
Cardiovascular death	Registrar General of Ontario Database[a]	ICD-10 I00-I99
Myocardial infarction[b]	Canadian Institute for Health Information Discharge Abstract Database (CIHI DAD)	ICD-10-CA I21, I22
Stroke[b]	CIHI DAD	ICD-10-CA I60, I61, I63 (excluding I63.6), I64, H34.1
Heart failure[b]	CIHI DAD	ICD-10-CA I50
Coronary revascularization[b] (percutaneous coronary intervention, coronary artery bypass graft surgery)	CIHI DAD	CCI 1IJ50, 1IJ54, 1IJ57GQ, 1IJ76

Abbreviations: CCI, Canadian Classification of Health Interventions; ICD, International Classification of Diseases.

[a] Contains information from the death certificates of all deaths registered in Ontario since 1990.

[b] The database captures information on demographic, administrative, clinical information on patients who are hospitalized to any acute care hospitals in Ontario. Abstractors captured information using ICD codes as instructed by the manual.

© 2019 Ko DT et al. *JAMA Network Open.*

eTable 2. Classification of Physicians Into Specialty Groups

Main specialty in ICES Physician Database	Specialty groups
Anatomical pathology	Laboratory physician
Anesthesiology	Anesthesiologist
Cardiac surgery	Surgeon
Cardiology	Cardiologist
Clinical immunology	Internist
Community medicine/public health	Family physician
Critical care	Internist
Dermatology	Internist
Diagnostic radiology	Radiologist
Emergency medicine	Internist
Endocrinology	Internist
Family practice/emergency medicine	Family physician
Gastroenterology	Internist
General pathology	Laboratory physician
General surgery	Surgeon
Geriatric medicine	Internist
General practice/family practice	Family physician
Hematological pathology	Laboratory physician
Hematology	Internist
Infectious diseases	Internist
Internal medicine	Internist
Laboratory medicine	Laboratory physician
Medical biochemistry	Laboratory physician
Medical genetics	Laboratory physician
Medical microbiology	Laboratory physician
Medical oncology	Internist
Nephrology	Internist
Neurology	Internist
Neuropathology	Laboratory physician
Neurosurgery	Surgeon
Nuclear medicine	Radiologist
Obstetrics and gynecology	Obstetrician/Gynecologist
Occupational medicine	Internist
Ophthalmology	Surgeon
Orthopedic surgery	Surgeon
Otolaryngology	Surgeon
Paediatrics	Paediatrician
Pediatric cardiology	Paediatrician
Pediatric clinical immunology	Paediatrician
Pediatric critical care	Paediatrician

© 2019 Ko DT et al. *JAMA Network Open.*

Pediatric endocrinology	Paediatrician
Pediatric gastroenterology	Paediatrician
Pediatric hematology	Paediatrician
Pediatric infectious diseases	Paediatrician
Pediatric nephrology	Paediatrician
Pediatric neurology	Paediatrician
Pediatric respirology	Paediatrician
Pediatric rheumatology	Paediatrician
Pediatric surgery	Paediatrician
Pediatrics	Paediatrician
Physical medicine and rehabilitation	Internist
Plastic surgery	Surgeon
Psychiatry	Psychiatrist
Radiation oncology	Internist
Respirology	Internist
Rheumatology	Internist
Thoracic surgery	Surgeon
Urology	Surgeon
Vascular surgery	Surgeon

© 2019 Ko DT et al. *JAMA Network Open.*

eTable 3. Crude Rates of Use of Ambulatory Health Services Among Physicians and the General Population

	Physicians (N=17,071)	General population (N=5,306,038)	P value
	n (%), unless otherwise stated		
**Physician visits**			
Primary care physician	14,739 (86.3)	4,965,617 (93.6)	<.001
Mean annual number of visits (SD)	1.55 (2.71)	3.98 (4.13)	<.001
Any specialist	15,866 (92.9)	4,528,776 (85.4)	<.001
Mean annual number of visits (SD)	2.81 (4.48)	2.16 (3.08)	<.001
Cardiologist	4318 (25.3)	1,027,364 (19.4)	<.001
**Periodic health exam**	9,352 (54.8)	3,599,582 (67.8)	<.001
**Cardiac risk factor assessment**[a]			
Cholesterol screening	12,792 (74.9)	4,443,488 (83.7)	<.001
Diabetes screening	12,510 (77.1)	4,003,644 (85.1)	<.001
**Cardiac testing**			
Electrocardiography	10,931 (64.0)	3,833,912 (72.3)	<.001
Echocardiography	5763 (33.8)	1,705,835 (32.1)	<.001
Non-invasive stress test	5056 (29.6)	1,569,171 (29.6)	0.9

Abbreviations: SD, standard deviation.
[a] Cholesterol screening calculated among all individuals ≥ 40 years as recommended by the Canadian Cardiovascular Society. Diabetes screening calculated among all non-diabetics at baseline and excluded testing during pregnancy.

© 2019 Ko DT et al. *JAMA Network Open.*

eTable 4. Use of Ambulatory Health Services Among Physicians and the General Population, Stratified by Sex, 2008-2015[a]

	MEN			WOMEN		
	Physicians	General population	P value	Physicians	General population	P value
	% (95 CI)			% (95 CI)		
**Physician visits**	**(N=17 071)**	**(N=5 306 038)**		**(N=17 071)**	**(N=5 306 038)**	
Primary care physician	83.4 (81.7, 85.1)	92.3 (92.2, 92.5)	<.001	92.6 (89.7, 95.7)	94.8 (94.7, 94.9)	0.16
Mean annual number of visits	1.4 (1.3, 1.4)	3.6 (3.6, 3.6)	<.001	2.1 (2.0, 2.2)	4.4 (4.4, 4.4)	<.001
Specialists	91.8 (90.1, 93.6)	82.8 (82.6, 82.9)	<.001	95.2 (92.2, 98.3)	88.0 (87.9, 88.1)	<.001
Mean annual number of visits	2.5 (2.5, 2.6)	2.0 (2.0, 2.0)	<.001	3.5 (3.3, 3.6)	2.3 (2.3, 2.3)	<.001
Cardiologists	27.3 (26.4, 28.2)	20.9 (20.9, 21.0)	<.001	23.3 (21.5, 25.1)	18.1 (18.0, 18.1)	<.001
**Periodic health exam**	48.0 (46.8, 49.3)	63.0 (62.9, 63.1)	<.001	69.3 (66.7, 71.9)	72.5 (72.4, 72.6)	0.017
**Cardiac risk factor assessment[b]**						
Cholesterol screening	72.9 (71.3, 74.4)	81.2 (81.1, 81.3)	<.001	79.6 (76.8, 82.5)	86.3 (86.2, 86.4)	<.001
Diabetes screening	74.7 (73.1, 76.4)	82.2 (82.1, 82.3)	<.001	83.1 (80.2, 86.1)	88.2 (88.1, 88.3)	<.001
**Cardiac testing**						
Electrocardiography	64.8 (63.4, 66.3)	71.7 (71.6, 71.8)	<.001	63.9 (61.3, 66.6)	73.0 (72.9, 73.1)	<.001
Echocardiography	35.4 (34.3, 36.5)	32.6 (32.5, 32.7)	<.001	32.7 (30.7, 34.7)	32.0 (31.9, 32.1)	0.53
Non-invasive stress test	31.6 (30.6, 32.7)	30.8 (30.7, 30.9)	0.10	26.6 (24.9, 28.5)	28.6 (28.6, 28.7)	0.029

Abbreviations: CI, confidence interval.
[a] Rates were age-standardized to the 2006 Ontario Census population.
[b] Cholesterol screening is calculated among all individuals ≥ 40 years as recommended by the Canadian Cardiovascular Society. Diabetes screening is calculated among all non-diabetics at baseline and excluded testing during pregnancy.

© 2019 Ko DT et al. *JAMA Network Open.*

eTable 5. Rates of Major Adverse Cardiovascular Events Among Physician Subgroups vs the General Population[a]

Subgroup	No. of physicians	No. of general population	Crude rate per 1000 person-years		p-value
			Physicians	General population	
**All participants**	17 071	5 306 038	4.5	6.2	<.001
**Age, y**					
40-54	9975	3 060 591	2.3	3.2	<.001
55-64	4925	1 382 845	5.8	7.9	<.001
65-75	2171	862 602	12.6	15.1	0.01
**Sex**					
Male	11 963	2 556 044	5.9	8.6	<.001
Female	5108	2 749 994	1.4	4.0	<.001
**Neighborhood income quintile**					
1 (lowest)	1218	967 939	4.8	7.4	0.003
2	1067	1035 605	4.6	6.7	0.02
3	1635	1 045 829	5.2	6.2	0.15
4	2725	1 101 501	4.3	5.7	0.005
5 (highest)	10,380	1 137 565	4.5	5.2	0.005
**Specialty**					
Anesthesiology	782	NA	3.1	6.2	0.003
Cardiology	400	NA	4.5	6.2	0.24
Family medicine	8018	NA	4.6	6.2	<.001
Internal medicine (non-cardiology)	2242	NA	4.5	6.2	0.006
Laboratory medicine	430	NA	3.9	6.2	0.10
Obstetrics or gynecology	527	NA	4.9	6.2	0.29

© 2019 Ko DT et al. *JAMA Network Open.*

Pediatrics	794	NA	3.0	6.2	0.002
Psychiatry	1480	NA	4.9	6.2	0.08
Radiology	671	NA	5.0	6.2	0.28
Surgery	1705	NA	5.4	6.2	0.24

Abbreviations: NA, not applicable; No, number.

[a] Major adverse cardiovascular events consist of acute myocardial infarction, stroke, heart failure, percutaneous coronary intervention, coronary artery bypass grafting or cardiovascular death.

© 2019 Ko DT et al. *JAMA Network Open.*

eTable 6. Sequential Adjustment to Explore Factors Associated With Risk of a Major Adverse Cardiovascular Event[a]

Variables	Additional adjustment for:				
	Age, sex	+ Income quintile	+ Traditional risk factors	+ Johns Hopkins ACG score	+ Health services
	Hazard ratio (95% CI)				
**Physician (Non-physician as reference)**	0.62	0.68	0.78	0.80	0.78
	(0.57, 0.67)	(0.63, 0.74)	(0.72, 0.85)	(0.74, 0.87)	(0.72, 0.85)
**Age on Jan 1, 2008**	1.08	1.08	1.07	1.07	1.07
	(1.08, 1.08)	(1.08, 1.08)	(1.07, 1.07)	(1.07, 1.07)	(1.06, 1.07)
**Female (Male as reference)**	0.43	0.43	0.47	0.46	0.46
	(0.43, 0.43)	(0.43, 0.43)	(0.47, 0.48)	(0.45, 0.46)	(0.45, 0.46)
**Neighbourhood income quintile (highest quintile 5 as reference):**					
1		1.48	1.30	1.31	1.32
		(1.46, 1.49)	(1.29, 1.32)	(1.30 1.33)	(1.30, 1.33)
2		1.29	1.17	1.18	1.19
		(1.28, 1.31)	(1.16, 1.19)	(1.17, 1.20)	(1.17,1.20)
3		1.21	1.13	1.13	1.14
		(1.20, 1.23)	(1.11, 1.14)	(1.12, 1.15)	(1.12, 1.15)
4		1.13	1.08	1.08	1.08
		(1.12, 1.14)	(1.06, 1.09)	(1.07, 1.09)	(1.07, 1.10)
**Smoking**			1.52	1.50	1.50
			(1.49, 1.55)	(1.47, 1.53)	(1.48, 1.53)
**Total cholesterol, per 10 mg/dL**			1.02	1.02	1.02
			(1.02, 1.02)	(1.02, 1.02)	(1.02, 1.02)
**HDL, per 10 mg/dL**			0.93	0.93	0.93
			(0.93, 0.93)	(0.93, 0.93)	(0.93, 0.93)
**Hypertension**			1.56	1.50	1.50
			(1.54, 1.57)	(1.48, 1.51)	(1.48, 1.51)
**Diabetes**			1.72	1.69	1.67
			(1.70, 1.74)	(1.67, 1.71)	(1.65, 1.69)
**Johns Hopkins ACG score**				1.02	1.02
				(1.02, 1.03)	(1.01, 1.02)
**Mean annual family physician visits**					1.00
					(1.00, 1.00)
**Mean annual specialty physician visits (2006-07)**					1.02
					(1.02, 1.02)

Abbreviations: ACG, Adjusted Clinical Group; CI, confidence interval; HDL, high density lipoprotein cholesterol.

[a] Major adverse cardiovascular events consist of acute myocardial infarction, stroke, heart failure, percutaneous coronary intervention, coronary artery bypass grafting or cardiovascular death

© 2019 Ko DT et al. *JAMA Network Open.*
